# Author Correction: Mapping disease regulatory circuits at cell-type resolution from single-cell multiomics data

**DOI:** 10.1038/s43588-023-00523-1

**Published:** 2023-08-31

**Authors:** Xi Chen, Yuan Wang, Antonio Cappuccio, Wan-Sze Cheng, Frederique Ruf Zamojski, Venugopalan D. Nair, Clare M. Miller, Aliza B. Rubenstein, German Nudelman, Alicja Tadych, Chandra L. Theesfeld, Alexandria Vornholt, Mary-Catherine George, Felicia Ruffin, Michael Dagher, Daniel G. Chawla, Alessandra Soares-Schanoski, Rachel R. Spurbeck, Lishomwa C. Ndhlovu, Robert Sebra, Steven H. Kleinstein, Andrew G. Letizia, Irene Ramos, Vance G. Fowler, Christopher W. Woods, Elena Zaslavsky, Olga G. Troyanskaya, Stuart C. Sealfon

**Affiliations:** 1 Center for Computational Biology, Flatiron Institute, New York, NY USA; 2 Lewis-Sigler Institute of Integrative Genomics, Princeton University, Princeton, NJ USA; 3 Department of Computer Science, Princeton University, Princeton, NJ USA; 4 Department of Neurology, Icahn School of Medicine at Mount Sinai, New York, NY USA; 5 Division of Infectious Diseases, Department of Medicine, Duke University School of Medicine, Durham, NC USA; 6 Program in Computational Biology and Bioinformatics, Yale University, New Haven, CT USA; 7 Battelle Memorial Institute, Columbus, OH USA; 8 Division of Infectious Diseases, Department of Medicine, Weill Cornell Medicine, New York, NY USA; 9 Department of Genetics and Genomic Sciences, Icahn School of Medicine at Mount Sinai, New York, NY USA; 10 Department of Pathology and Department of Immunobiology, Yale School of Medicine, New Haven, CT USA; 11 Naval Medical Research Center, Silver Spring, MD USA; 12 Precision Immunology Institute, Icahn School of Medicine at Mount Sinai, New York, NY USA

**Keywords:** Computational models, Epigenomics

Correction to: *Nature Computational Science* 10.1038/s43588-023-00476-5. Published online 25 July 2023.

In the version of this article originally published, Elena Zaslavsky and Olga G. Troyanskaya were not listed as corresponding authors, and in the Acknowledgements, NIAID grant UM1AI164559 was shown incorrectly. The errors have been corrected in the HTML and PDF versions of the article.

